# A 3D View of Colorectal Cancer Models in Predicting Therapeutic Responses and Resistance

**DOI:** 10.3390/cancers13020227

**Published:** 2021-01-10

**Authors:** Eileen Reidy, Niamh A. Leonard, Oliver Treacy, Aideen E. Ryan

**Affiliations:** 1 Lambe Institute for Translational research, School of Medicine, College of Medicine, Nursing and Health Sciences, National University of Ireland Galway, H91 V4AY Galway, Ireland; e.reidy3@nuigalway.ie (E.R.); n.leonard4@nuigalway.ie (N.A.L.); oliver.treacy@nuigalway.ie (O.T.); 2 Regenerative Medicine Institute (REMEDI), School of Medicine, College of Medicine, Nursing and Health Sciences, National University of Ireland Galway, H91 W2TY Galway, Ireland; 3 Discipline of Pharmacology and Therapeutics, School of Medicine, College of Medicine, Nursing and Health Sciences, National University of Ireland Galway, H91 W5P7 Galway, Ireland; 4 CÚRAM, SFI Research Centre for Medical Devices, NUI Galway, H91 W2TY Galway, Ireland

**Keywords:** 3D models, colorectal cancer, drug resistance, spheroids, organoids, microfluidic devices, tumor microenvironment, drug development

## Abstract

**Simple Summary:**

Colorectal cancer is one of the most common solid tumors in the developed world. Although there have being many advances in treatment options in recent years, many patients develop resistance to treatment which impacts their outcome. It has been shown that cancer cells can interact with cells around them in the colon to help the tumor to progress, expand, and resist death in response to treatment. To study how the cancer cells interact with the different cells in the colon, 3D models can be used. They allow many different cell types to be incorporated together and investigation of their response to drugs over time. This review aims to summarize the advantages and disadvantages of 3D models currently being used to study colorectal cancer, as well as suggesting how these models could be useful in studying drug resistance and the development of new drugs.

**Abstract:**

Although there have been many advances in recent years for the treatment of colorectal cancer (CRC), it still remains the third most common cause of cancer-related deaths worldwide. Many patients with late stage CRC display resistance to multiple different therapeutics. An important aspect in developing effective therapeutics for CRC patients is understanding the interactions that take place in the tumor microenvironment (TME), as it has been shown to contribute to drug resistance in vivo. Much research over the past 100 years has focused on 2D monolayer cultures or in vivo studies, however, the efficacy in translating these to the clinic is very low. More recent studies are turning towards developing an effective 3D model of CRC that is clinically relevant, that can recapitulate the TME in vitro and bridge the gap between 2D cultures and in vivo studies, with the aim of reducing the use of animal models in the future. This review summarises the advantages and limitations of different 3D CRC models. It emphasizes how different 3D models may be optimised to study cellular and extracellular interactions that take place in the TME of CRC in an effort to allow the development of more translatable effective treatment options for patients.

## 1. Introduction

Colorectal cancer (CRC) is the third most common cause of cancer-related deaths worldwide [1] and develops, primarily, as a result of mutations that target oncogenes, tumor suppressor genes and genes related to DNA repair mechanisms. These mutations inform the classification of CRC as sporadic (70%), familial (25%) or inherited (5%) [2]. The incidents of CRC has been steadily increasing worldwide, with a worrying increasing rate of diagnosis of younger patients (<50 years old) [3]. Key risk factors associated with the development of CRC include consumption of red meat, age, stress, smoking, alcohol consumption and obesity [3,4,5,6]. Chronic inflammation is linked to the development of CRC and diseases such as inflammatory bowel disease and diabetes have been associated with increased risk. [7,8,9,10,11]. In addition to risk factors, individuals can be predisposed to the development of CRC due to inherited diseases such as such as Lynch syndrome or familial adenomatous polyposis [7]. In early-stage CRC, surgery is the most successful treatment modality, however, many patients present at diagnosis with metastatic disease and surgery is no longer an option [12]. Although new therapeutic options are becoming available for CRC patients, the number of overall deaths rises each year [13]. Like most cancers, there are multiple different mutations associated with CRC and, as a result, no one specific molecular therapy is effective for all patients [14]. Additionally, it has being shown that over 90% of patients diagnosed with late stage CRC are resistant to frontline treatments [15].

Guinney et al. [16] proposed a consensus on classifying the different molecular subtypes of CRC based on RNA expression profiling. Consensus molecular subtypes (CMS) are associated with different mutations, levels of immune infiltration, and different somatic copy number alterations. CMS1 is associated with microsatellite instable, high immune infiltration tumors, CMS2 is associated with chromosomally instable CRC, CMS3 is associated with metabolic dysregulation, and CMS4, which accounts for 23% of all CRC cases, is the subtype that is associated with worse overall survival [16]. Patients with CMS4 CRC have high stromal infiltration, a strong immune signature and increased angiogenesis, making it difficult to treat and outlining the effect the tumor microenvironment (TME) as a whole can have on treatment response and outcome [16]. Understanding the interactions that take place between CRC cells, stromal cells and other immune cells could potentially lead to the development of new drugs or treatment schedules and immunotherapeutic treatments for patients with CRC.

In vivo models have previously been used to investigate the complex multicellular components of tumors [17,18]. Although these models have many advantages over in vitro studies, there are still a number of limitations to this approach including the cost of animal models, the number of animals needed for time-point experiments [19], sensitivity of cell imaging in vivo and animal ethics [20]. Another limitation is that species difference can mean therapeutics are often not translatable to humans [21]. 2D cell culture is one of the most widely used techniques for studying CRC cells in vitro. However, recent studies have shown that 2D culture does not accurately represent the cellular and matrix interactions that take place in vivo [20].

**Figure 1:**
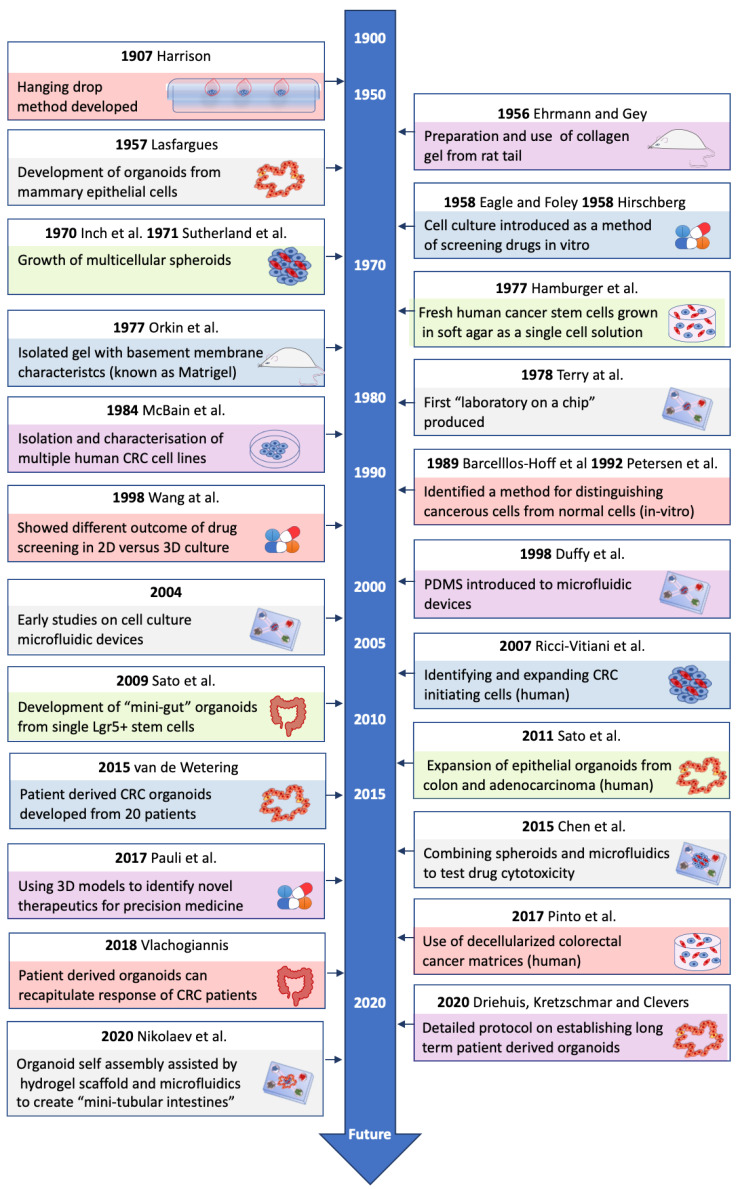
Timeline of major advances in 3D models of colorectal cancer (CRC). This figure summarises some of the major advances in the development of different 3D models. It includes some of the major findings that have led to the development of current 3D models of CRC [27,28,29,30,31,32,33,34,35,36,37,38,39,40,41,42,43,44,45,46,47,48,49,50,51].

3D models have emerged in recent years as tumor models that are more physiologically relevant than 2D models [22]. Cells grown in 3D more closely resemble in vivo conditions in terms of cell morphology, protein expression, biomarker expression and gene expression [23,24]. There have been many advances in the development of 3D CRC models over the years (see Figure 1). 3D models have also been shown to be valuable in drug discovery [25], analyzing drug resistance, as well as studying cell-cell and cell-matrix interactions that occur in the TME of CRC [20]. Co-culture 3D models, introducing stromal or immune cells, have provided knowledge about the roles these cells play in the TME [22,26]. As shown by Guinney and colleagues [16], the stromal compartment of CRC tumors can alter disease progression and response to therapy, which could be a key area that 3D cultures may help understand and aid development of new treatment options.

This review aims to summarize the different types of 3D models currently being used to study CRC. It outlines in detail the advantages and limitations of each model and how these 3D models incorporate different components of the TME to mimic in vivo CRC. Finally, we show how 3D models may be able to help in determining drug resistance to new therapeutics in the future.

## 2. Tumor Microenvironment

A more complete understanding of the interactions that take place between cells in the CRC TME could lead to the development of new therapeutics for improving the outcome for patients. In addition to the cancer cells, the TME primarily consists of tumor-infiltrating immune cells, mesenchymal stromal cells (MSCs), cancer associated fibroblasts (CAFs), the extracellular matrix (ECM), and vasculature [52,53,54,55,56]. Many of the limitations of current treatments are that interactions between these components are not fully understood [57]. It is essential to study what interactions contribute to tumor progression in order to develop effective therapeutics for the treatment of CRC.

Mesenchymal cells are an important cellular component in the TME and can be recruited as resident MSCs and bone marrow MSCs [55,58,59,60,61]. They include multiple cell types including MSCs, fibroblasts, smooth muscle cells, and pericytes which each have diverse subtypes expressing a variety of different cell markers [53,59,60]. Upon activation, these cells lead to the development of CAFs [53,56,59]. Although the interactions that take place between MSCs and CRC cells are not fully understood, high stromal infiltration has been shown to be associated with worse overall survival in CRC patients [16]. One of the factors that is thought to contribute to this is the secretion of growth factors such as fibroblast growth factor or hepatocyte growth factor by both MSCs and CAFs, which can promote tumor growth [62]. Furthermore, CAFs promote metastasis through secretion of transforming growth factor-beta (TGF-β), leading to an immunosuppressive environment [63] and have also been shown to contribute to resistance to conventional therapies such as chemotherapy [64]. MSCs can alter the anti-tumor immune response and enable tumor progression through expression of immunomodulatory ligands, such as programmed death-ligand 1 (PD-L1), PD-L2, and Fas ligand [17,65]. Expression of immunomodulatory ligands on stromal cells in the TME could be important in stratifying patients for immunotherapy [17]. For this reason, it is important to develop models of CRC that can be used to study interactions that take place between stromal and CRC cells. This could also potentially help in determining what treatments CRC patients may be resistant to and identifying combination therapies that may overcome this.

**Figure 2:**
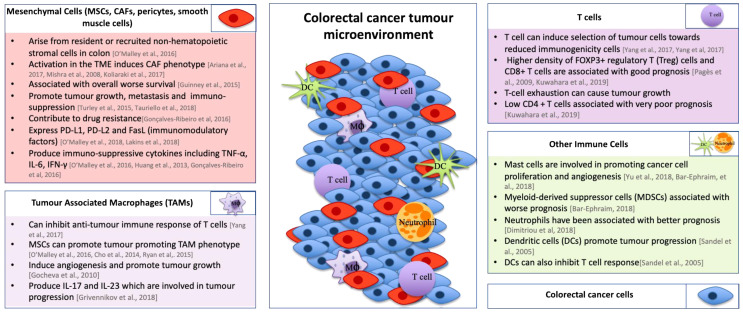
The role of various cells in the tumor microenvironment of colorectal cancer. The diagram in the centre of this figure represents the tumor microenvironment (TME) of CRC. This figure summarises the role that different cells play in the TME of CRC [16,17,55,56,59,60,61,62,63,64,65,68,69,77,78,79,80,82,83,84,85,86,87,88].

The immune infiltrate in CRC can vary between subtypes and can include a number of different immune cell subtypes including T-cells, neutrophils, monocytes, natural killer cells, mast cells, and endothelial cells [66,67]. Under normal circumstances, the immune system has the ability to eliminate cancer cells. Tumors have developed strategies to overcome this by polarizing immune cells to a tumor-promoting phenotype [68,69,70,71]. One particularly important example is tumor-associated macrophages (TAMs). The role of TAMs in the TME of CRC is often controversial as they have been reported to have both positive and negative effects on patient survival [72,73]. However, high numbers of TAMs have correlated with worse prognosis in over 80% of human cancers [74,75]. Multiple papers have shown that TAMs can lead to genic instability in cancer cells [76], induce angiogenesis, and promote tumor growth and contribute to ECM degradation [68,74]. MSCs have been shown to promote TAM polarisation towards a tumor-promoting phenotype through production of IL-10 and decrease in secretion of inducible nitric oxide synthase (iNOS) and IL-12 [52,76,77]. TAMs can also inhibit T-cell anti-tumor immune responses [77] and have been shown to contribute to chemoresistance through production of IL-6 [78]. T-cells play critical roles in the adaptive immune response. High levels of CD8+ T-cells and forkhead box P3+ (Foxp3+) regulatory T (Treg)-cells have been linked to a better prognosis in CRC patients [79,80]. However, contradictorily, Foxp3+ T-cells, as well as CD4+ T-cells, have been associated with very poor prognosis in CRC patients [80]. Mast cells have also been shown to be involved in promoting cancer cell proliferation and angiogenesis at various stages of CRC development [69]. Hypoxic regions in the TME affect angiogenesis through activation of hypoxia inducible factor-1α (HIF-1α), which regulates expression of angiogenic factors such as vascular endothelial growth factor, further promoting angiogenesis leading to a more aggressive tumor phenotype [81]. Overall, the function of different immune cells present in the TME of CRC plays an important role in CRC progression (see Figure 2).

In addition to the cellular components of the TME, the ECM also has a key role to play in CRC. It provides cells with structural support, allowing for cell proliferation, growth and migration to take place [53]. The main components of ECM include structural proteins such as collagen, fibronectin, laminin, proteoglycans and hyaluronic acid [53,89,90,91,92]. The ECM also contains a variety of cytokines and growth factors which can affect the initiation and progression of CRC [93]. Previous studies have demonstrated that the ECM of CRC changes dynamically in each stage of development, showing its importance in influencing cancer progression [89]. As well as changes in composition of the ECM, the stiffness of the ECM increases as CRC progresses [94] and increased stiffness may reduce drug delivery and promote resistance [95]. Therefore, an ECM-based model or a model combining cells which secrete ECM proteins or incorporate ECM in their design would be advantageous. Models that replicate the TME, with a stiffness comparable to the in vivo environment, may more accurately represent the TME of CRC than 2D cell culture. As the communication between multiple cell types in the TME dictate tumor progression, these 3D cultures may allow for the development of novel treatments focused on targeting the immunosuppressive effects of different immune cells in the TME of CRC.

## 3. Current 3D Models of CRC

Much of our knowledge on CRC development and progression has been generated from immortalized cell lines. 2D cell culture involves growing cells on tissue culture plastic, thereby forming a monolayer of cells. The main advantages of 2D cultures are their low cost, ease to maintain in culture and the standardized culture techniques which are universally used and highly repeatable [96]. Another advantage of 2D culture is that different CRC cell lines can, to a certain extent, recapitulate CMS features and therefore can give an initial indication about how different treatments can affect CRC cells [97]. However, 2D cultures do not reflect the complexity of CRC and its microenvironment. Studies with monolayer cultures have shown that cells cultured in 2D respond differently to treatments [25], have differentially expressed proteins [24], have altered gene expression profiles, as well as altered intercellular signaling [23] compared to 3D cultures. Additionally, 2D models are limited in their potential to determine the contribution of other cells in the TME to the processes of progression, metastasis, immune evasion, and drug resistance. For these reasons, a number of 3D models have been developed that more closely represent CRC tumors in vivo. Many of these models can incorporate stromal cells, immune cells and even vasculature [19,23,95] to better mimic the in vivo microenvironment and include spheroids, organoids, and microfluidic devices.

### 3.1. Scaffold-Free Spheroids

**Figure 3:**
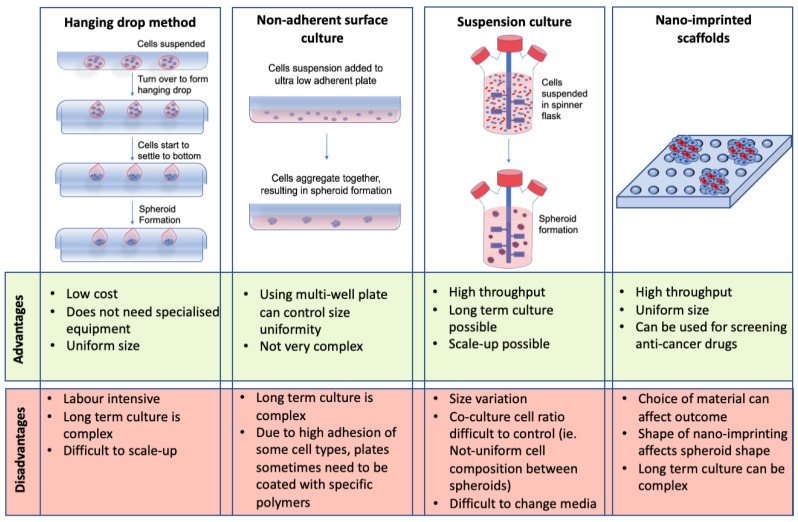
Advantages and disadvantages of spheroids as a 3D culture model. Different methods for spheroid formation, including their respective advantages and limitations, are shown including the hanging drop method, non-adherent surface culture, suspension culture, and nano-imprinted scaffolds [98,99,100,101,102,103,104,105,106].

Spheroids are aggregates of cells that grow in 3D. They are designed to more closely resemble in vivo models compared to their 2D culture counterparts. CRC spheroids represent a 3D avascular model of CRC that encapsulates cell-cell and cell-matrix interactions [20,25]. Some of the main methods for scaffold-free spheroid formation include the hanging drop method, non-adherent surface culture, suspension culture, and nano-imprinted scaffolds [98,99,100,101]. Although these are some of the most commonly used methods for spheroid formation, they all have different advantages and limitations (see Figure 3).

Despite the limitations discussed in Figure 3, CRC spheroids have been successfully used to study tumor growth, proliferation, invasion [98,107], micro-metastasis [98], immune cell interactions [17,22], as well as a drug screening tool [17,19,22]. Gene expression analysis has also been performed on CRC spheroids containing hypoxic and necrotic regions and it was found that these spheroids mimic the gene expression profile of in vivo tumors [108]. Although studies have been carried out developing spheroids with CRC cells only [109], these spheroids do not allow for the exploration of the complex TME. Spheroids incorporating stromal and immune cells more accurately represent CRC in vivo [22,26]. Studies have shown that incorporating stromal cells into CRC spheroids alter specific pathway expression in the co-cultures versus the mono-culture spheroids. These include the Ras and nuclear factor-kappa B (NF-κB) signaling pathways [26]. NF-κB is associated with inflammation and CRC progression [87,110], demonstrating the importance of incorporating stromal cells into CRC spheroids to mimic the in vivo microenvironment.

In recent years, CRC spheroids have been developed using patient-derived primary cancer cells. The use of individual patient samples further increases the likelihood of identifying translatable targets as spheroids using primary cells can histologically resemble original patient tumors and show similar protein expression patterns to the tumor in vivo [111]. CRC spheroids can also test the therapeutic potential of multiple cancer treatments including immunomodulatory antibodies targeting major histocompatibility complex class I chain-related protein A and Natural killer(NK) group 2 member A [20], as well as combination therapies involving T-cell bispecific antibodies with an interleukin-2-variant [22]. Studies using spheroids derived from different CRC cell lines with the addition of stromal cells have also been used to study combination therapies including 5-fluorouracil, erlotinib, and regorafenib [25]. These studies show that spheroids can be used as a clinically relevant model of CRC for testing treatments in a 3D microenvironment.

### 3.2. Hydrogels

#### 3.2.1. Natural Hydrogels

Another method for developing 3D models of CRC is to use hydrogels. Hydrogels are ECM-like materials that provide cells with a biocompatible substance in which they can grow and proliferate in 3D-forming spheroids. They can be composed of natural or synthetic material. Natural hydrogels are derived from plants and animal materials which are highly biocompatible. Some of the most frequently used natural hydrogels include collagen type I, Matrigel, gelatin, and alginate. Others such as hyaluronic acid, agarose, and chitosan are also commonly used [112,113,114,115].

Collagen is the most abundant protein present in the ECM of animals and it is present in the TME at different levels at each stage of CRC [89]. It plays a role in cell signaling, tumor progression, metastasis, and cell migration [112]. Collagen type I hydrogels are often used in the development of 3D models of CRC due to their ability to provide an ECM-like structure [116,117]. They are often prepared through altering the pH or temperature of the acidic gel [116], resulting in an ECM-like matrix. These hydrogels have been shown to provide structural support to cells allowing them to grow in 3D in order for cell–cell and cell–matrix interactions to be observed.

Collagen-embedded CRC models have been used to study invasion. A recent study created 3D models of CRC with a stromal surround [118]. Through quantitative image analysis and analysis of angiogenic factors and expression of invasive markers (matrix metalloproteinase-7 (MMP7) and heparinase), it was found that the complexity of stroma within the CRC spheroids directly affected the aggressiveness of the CRC [118]. Under normal conditions, the stiffness of the colon is typically around 0.936 kPa, however, as CRC starts to progress the tissue stiffness can range from 2.81 kPa to 13.8 kPa [94]. Crosslinkers such as lysyl oxidase (LOX) increase collagen matrix stiffness and provide a more physiologically relevant matrix [117]. LOX-like enzymes can increase stiffness of the scaffold which in turn was shown to increase CRC cell migration [117]. RAFT absorption kits have also been used to remove any excess fluid present in the hydrogel model. This leads to an increase in density of the collagen matrix, making it more physiologically relevant and promoting cell migration and micro-metastasis [116]. Both of these studies show that the stiffness of the hydrogel ECM can alter cell activity in 3D models.

Matrigel is another common natural hydrogel used in the development of 3D models of CRC. It is composed of ECM material extracted from an Engelbreth–Holm–Swarm (EHS) mouse sarcoma containing proteins such as collagen IV and laminin [119], as well as a variety of different growth factors [120]. Often Matrigel is used to coat wells for transwell assays to study cell invasion [121], while it has also been used for preparing 3D cancer models. Chandrasekaran et al. [114], have used Matrigel 3D models to investigate the effects of different inhibitors on CRC cell invasion rate and metastasis, demonstrating a use for this hydrogel in 3D model formation. Although used in 3D models, Matrigel does not provide the stiffness needed to mimic the ECM in vivo. In order to overcome this limitation, it has been combined with collagen to increase stiffness [50]. Matrigel is notoriously variable between batches due to the presence of undefined material in the gel [122]. This can cause difficulty when comparing results of different experiments.

Alginate is an algae-derived polysaccharide that is non-toxic, highly biocompatible and, unlike collagen and Matrigel, a bio-inert substance [113,123]. The crosslinker for alginate is calcium chloride, whereby alginate hydrogels form stable structures through ionic interactions that take place between calcium cations (Ca^2+^) and G units of the polymer backbone forming an ‘egg box’ like structure [124]. Alginate beads containing HCT116 cells, a CRC cell line, have been previously used to demonstrate relative drug resistance and the effects of fluorouracil (5-FU) chemotherapy on CRC cells in 3D [125]. Treating HCT116 in alginate gels with paclitaxel showed a decrease in glucose uptake, cell proliferation and cell viability [126]. Both of these studies concluded that embedding CRC cells in alginate beads could be used to test chemotherapeutic drugs. However, developing 3D models using alginate has a number of limitations regarding biomechanical and bioactivity properties. In order to combat these problems, some studies have conjugated integrin-binding motif arginine, glycine, aspartic acid (RGD) to alginate in order to introduce cell-adhesive sites [127]. Other studies have shown that alginate can also be combined with other hydrogels such as gelatin to create a hydrogel that promotes cell adhesion, spreading of cells and proliferation [113].

Gelatin is a hydrogel that is derived from denaturing collagen type I [128]. It is a highly-biocompatible material with integrin binding sites which regulate the activity of cells in the 3D matrix [129]. However, gelatin has poor mechanical properties. For this reason, it is often combined with other hydrogels in 3D models [113] or crosslinked to improve its biomechanical properties. One example of this is gelatin methacryloyl (GelMA), which results in a biocompatible hydrogel with stiffness that can be modulated precisely following the incorporation of methacrylamide and methacrylate groups that can be crosslinked in the presence of a photoinitiator [130]. With the increasing stiffness of CRC with increasing disease stage, this could be a valuable model [94]. It can also be used for bioprinting, leading to the formation of more complex cancer models [131].

As well as GelMA, there are multiple other studies that have used modified natural hydrogels through introduction of peptides or crosslinkers. These modified natural hydrogels take the biocompatibility of natural hydrogels and combines them with the adjustability and reproducibility of synthetic hydrogels [132]. A study carried out by Magdeldin et al. [116], introduced laminin into collagen gels, promoting stromal cell growth in a vasculature-like structure, more closely mimicking how these cells act in vivo. Another study introduced RGD-binding motifs to alginate gels to more closely mimic the adhesion sites present in vivo [127]. Overall, modifying natural hydrogels allows for these gels to have biocompatible properties, while mimicking the ECM in vivo in terms of stiffness and binding sites for cells and may provide the ideal hybrid between natural and synthetic hydrogels.

#### 3.2.2. Synthetic Hydrogels

In addition to natural hydrogels, synthetic hydrogels can be used for the development of 3D cancer models. Synthetic hydrogels are polymers that can be altered to mimic certain aspects of the ECM in vivo. Examples include polyethylene glycol, macroporous hydrogels and polyvinylidene fluoride [133,134,135]. One of the main advantages of using synthetic hydrogels is that they are highly modifiable and customizable, meaning that they can often be manipulated to include integrin binding sites such as RGD binding motif, a binding site found in natural polymers [136]. Some hydrogels can also be manipulated to include MMP sites, which support growth of cells in a 3D microenvironment and also increases biodegradability [137].

**Figure 4:**
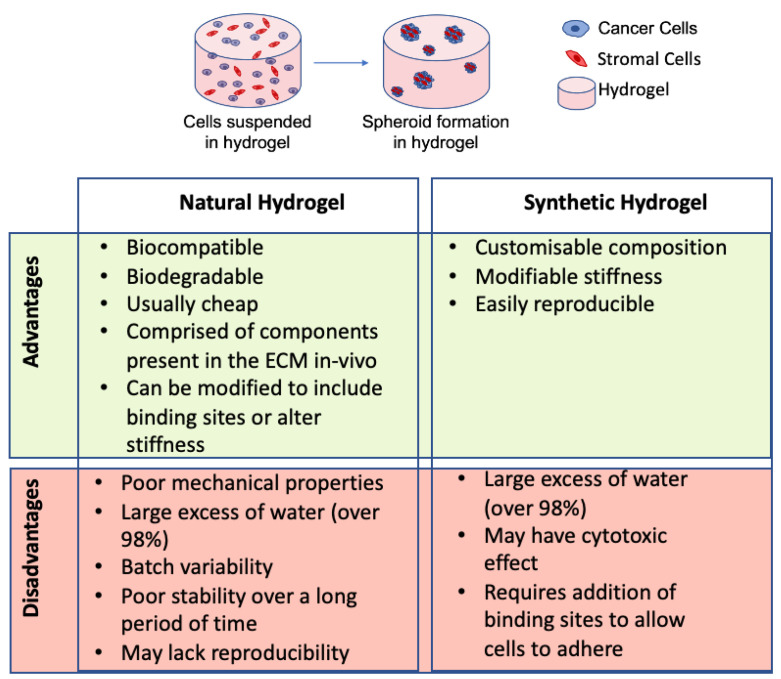
Advantages and disadvantages of natural and synthetic hydrogels. This diagram illustrates cells suspended in hydrogel and forming spheroids. This represents the use of hydrogels in the development of 3D models of CRC [116,122,132,134,136,138].

The stiffness of many synthetic hydrogels can also be altered to more closely mimic the stiffness of ECM in vivo [138]. Macroporous hydrogel scaffolds have been used to compare the effects of cisplatin on 2D versus 3D HCT116 cell culture [134]. It was found that cells in 3D were less susceptible to the effects of cisplatin treatment than cells grown in 2D, confirming the possible use of synthetic hydrogels in toxicity testing [134]. In general, synthetic hydrogels are not used as often as natural hydrogels for developing 3D models of CRC. This is due to the fact that they can often cause an immune response and often do not interact with cells causing poor cellular responses [139]. Furthermore, the synthetic gels usually have to be modified to include binding sites [136] and increase their biodegradability [137]. Depending on the purpose of the experiment, different hydrogels are more suitable for specific forms of research as both natural and synthetic hydrogels have advantages and disadvantages (see Figure 4).

### 3.3. Organoids

Another form of 3D culture that offers many advantages over 2D models is CRC organoids. Organoids are self-organised models, primarily derived from human pluoripotent stem cells (hPSCs) or adult multipotent stem cells. The primary difference is that multipotent stem cells are organ-specific whereas hPSCs can differentiate into multiple cell types including stromal and immune cells [140]. In recent years, there have been major advances in organoid development with a recent study using intestinal stem cells to produce self-assembling intestinal organoids, with crypt-like and villi-like regions, which resembled the spatial arrangement of these structures in vivo [50]. Organoids can be produced from individual patient tumor samples, meaning that they can provide similar biodiversity to in vivo tumors and could potentially be used for developing patient-specific treatments [48,140,141]. Previous studies have found that CRC organoids had 90% similarity in somatic mutations and 0.89 correlation with DNA copy number profiles between organoids and original patient biopsies [141]. These similarities emphasize the advantages of using organoids as a model of CRC.

CRC organoids have been used to study initiation, progression and invasion of CRC, as well as being used for drug screening [46,142]. Studies involving organoids have shown that introduction of mutations in genes coding for TGF-β, wingless-related integration site (Wnt), P53, and epidermal growth factor receptor (EGFR) promote tumor progression and metastasis [143]. Others have shown that introducing genetic mutations only led to micro-metastasis and chromosomal instability was needed to induce aggressive metastatic behaviour [144].

CRC organoids are also being used to look at the immunomodulatory properties of CRC. CRC organoids were co-cultured with cytotoxic T-cells to study the immunomodulatory properties of CRC, and observe the anti-tumor immune response of cytotoxic T-cells in vitro [70]. The role of leucine rich repeat containing G-protein coupled receptor 5 positive (Lgr5+) intestinal stem cells has also been analyzed using CRC organoids. It was found that Lgr5+ intestinal stem cells, progenitor cells of CRC, assist in tumor progression [145]. Many of these findings provide an insight into the interactions that take place in the TME and may identify targets for CRC therapeutics.

Organoids are often prepared in a Matrigel surround [146,147]. However, a recent study showed that collagen I is present in aggressive CRC [148]. This study replaced Matrigel with collagen I and found there was expression of tumor-specific mesenchymal genes and increased tumor invasion [148]. This highlights the effects of ECM on the behaviour of CRC;the interactions between collagen I and CRC cells may be important targets for developing treatments for aggressive CRC in the future.

Specific studies have been carried out to identify targets for treating CRC. High throughput drug screening analysis has been carried out using 19 organoid CRC lines to identify chemotherapeutic drugs and inhibitors of specific targets by screening 83 different drugs [46]. Other studies looked at the efficiency of chimeric antigen receptor (CAR)-engineered NK-92 cells as a therapy targeting ubiquitous epithelial cell adhesion molecule [149]. Another study analyzed similarities in chemoradiation response between rectal organoids and patients. Astonishingly, the responses matched with almost 85% accuracy and 92% specificity [150], showing the potential of organoids as a diagnostic tool for therapeutics.

Although organoids are used to study the efficacy of different treatments, they also have some limitations. It is more difficult to produce organoids from patients with mucinous tumors, microsatellite instable tumors, and tumors with a mutation in the BRAF gene, possibly indicating that organoids may not be the ideal 3D model for these tumor types [151]. Other studies have shown that the success rates of organoid development, even with significant expertise, is only around 70% [141]. Preparation of organoids requires access to a tissue network or hospital to obtain patient samples and requires expertise for organoid preparation and maintenance, adding to the limitations [46]. Another limitation of organoids and 3D models of CRC is that there is a lack of easy and reproducible readout methods. This can limit their uses in high throughput screening studies. However, overall, the use of specific organoids can be effective models for studying tumor progression, metastasis, and drug screening.

### 3.4. Microfluidic Devices

**Figure 5:**
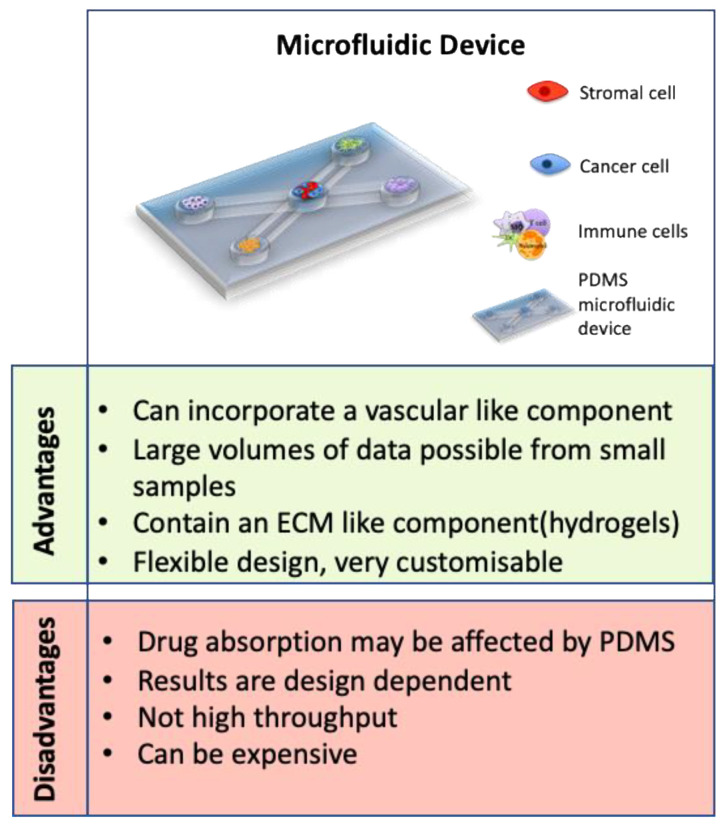
Advantages and disadvantages of microfluidic devices. A representative image of a microfluidic device is shown as well as a summary of the advantages and disadvantages of using a microfluidic device as a 3D model [154,156,157,158].

One of the most recent advances in 3D models is the development of microfluidic devices. Microfluidic devices are a technology that allow precise manipulation of minute liquid volumes through channels, as well as allowing compartmentalization of different cell types, offering scientists a platform to learn more about cancer and other disease models [152]. They are often prepared using polydimethylsiloxane (PDMS) molds and many incorporate hydrogels with multiple cell types [153,154]. The advantages of using microfluidic devices as 3D models of CRC vastly outweigh the limitations (see Figure 5). They have been used to study the role of vasculature, cancer progression and metastasis, as well as being used in drug development and diagnostics studies [95,154,155]. Interestingly, microfluidic devices are used to prepare heterogenic spheroids of a controllable size, containing cancer cells and stromal cells in a ratio of 1:1. By using time-lapse incorporated confocal imaging, this study successfully analysed metastasis of the CRC tumor spheroid and the interactions with stromal cells [155].

One of the limitations of other 3D models is that they often do not incorporate vasculature, which may affect the physiological relevance of these models. Some microfluidic devices are capable of overcoming this adversity by incorporating endothelial cells, mimicking the vasculature present in CRC in vivo [95,153,157]. A recent study developed a microfluidic device that contained HCT116 cells in Matrigel in the central chamber and incorporated human colonic microvascular endothelial cells in side channels to mimic vasculature [154]. After the addition of vascular endothelial growth factor receptor (VEGFR), endothelial cells started to invade the central chamber and form vasculaturebranches similar to early stages of CRC [154]. Another study co-cultured fibroblasts with the HT29 CRC cell line in a microfluidic device. This study reported migration of fibroblasts towards the chamber with HT29 cells. They found an increase in alpha smooth muscle actin (α-SMA) and filamentous actin expression upon activation of the fibroblasts. Furthermore, the authors found significant changes in multiple proteins associated with tumor progression including Serpin-E1, granulocyte macrophage-colony stimulating factor (GM-CSF), as well as an increase in angiogenic factors and a decrease in apoptosis-associated proteins [153]. These findings not only indicate the dynamic role of fibroblasts in tumor progression but also demonstrate the impact of vasculature inclusion in the system. This approach using microfluidics devices, incorporating multiple cellular components with ECM and fluid dynamics, more closely mimics the in vivo microenvironment of CRC.

In addition to their potential in diagnostics, microfluidic devices are used in drug screening treatments for CRC patients [159]. Microfluidic devices incorporating breast and colon cancer cells have been used to analyze vascular-targeting drugs including Apatinib, Vandetanib (targeting VEGFR only), Linifanib and Cabozantinib (targeting VEGFRs, PDGFR and Tie2). This study analyzed a vasculature-incorporating microfluidic device to determine that Linifanib and Cabozantinib caused regression of the vasculature, whereas the drugs targeting VEGFR alone did not, which could not have been determined using a traditional 2D culture [160]. Microfluidic devices have also been used to study the effects of Gemcitabine on CRC. Gemcitabine has been delivered to CRC cells using fluorescently-modified nanoparticles [154]. They were able to analyze the viability of cells in response to the treatment and also found the fluorescently-modified nanoparticles to be an effective tracking tool for analyzing the location of nanoparticles throughout the microfluidic device [154]. These studies further support the utility of microfluidic devices for analyzing the effectiveness of therapeutics in CRC in the future.

## 4. 3D Models for Determining Resistance to Therapeutics

Although there have been many advances in the development of models for studying CRC, patient survival rates still remain poor, with the 5-year survival rates for patients with late stage CRC at only 14% [161]. For many patients, especially those with advanced CRC, resistance to therapy is a significant challenge, with studies showing that 90% of patients with late stage CRC are resistant to frontline treatments [15] with almost half of all CRC patients resistant to 5-FU [162].

Over 60 years ago, 5-FU was introduced as a chemotherapeutic treatment for many types of cancer [163]. It is often combined with other chemotherapeutic treatments such as Oxaliplatin and Irinotecan, however, patients can develop resistance to these drugs [164]. 5-FU functions by inhibiting thymidylate synthase through replacement of thymidine in DNA with fluorinated nucleotides, which in turn inhibits DNA replication causing cell death [165]. The success of 5-FU in triggering cell death is highly dependent on expression of a number of enzymes including thymidine phosphorylase and dihydro-pyrimidine dehydrogenase, which contribute to the degradation and metabolism of 5-FU, leading to drug resistance [166,167]. Other mechanisms contributing to resistance to 5-FU include an increased level of DNA repair [165]. Furthermore, mechanisms of resistance vary for different chemotherapeutic drugs. For example, epigenetics and downregulation of topoisomerase 1 plays a role in resistance to Irinotecan [168] and accumulation of platinum is involved in resistance to Oxaliplatin [169]. The wide variety of resistance mechanisms, combined with resistance to different chemotherapeutics, emphasizes the need to develop an accurate 3D model that faithfully mimics the CRC microenvironment in vivo to test drug efficacy prior to clinical trials.

2D models used to test drug efficacy can recapitulate a direct anti-tumor response to chemotherapy, however, there is a need for a more clinically relevant model that can reveal effects on cells in the TME. It is estimated that 96.6% of all drugs in clinical trials for oncological purposes do not get approval by the United States Food and Drug Administration [170]. This emphasizes the need for more effective prediction models for testing these drugs as therapeutics for CRC. To date, studies using 3D models have investigated the effect of different chemotherapeutics on CRC including 5-FU, regorafenib and erlotinib [25]. Different CRC cell lines were co-cultured with fibroblasts and endothelial cells and changes in metabolic activity and spheroid size based on treatment were observed. The effects on 2D models and 3D co-culture models were analyzed. After testing the different cultures with Erlotinib, it was found that the 3D models showed a dose-dependent sensitivity to the drug in comparison to the 2D culture. The addition of fibroblasts and endothelial cells into the co-culture increased the resistance of the cultures to various combinatorial treatments. Another important aspect of this study was that the authors showed that the 3D model with and without co-culture responded differently to treatments, demonstrating the importance and urgent need for a reproducible multi-cellular 3D platform [25].

3D cultures provide cells with a more structured environment to proliferate and spheroids contain a variety of different cellular phenotypes including quiescent cells, proliferating cells, and necrotic cells, which may be responsible for how the cells respond differently to treatment in 2D versus 3D culture [108]. In CRC, regions of hypoxia have been shown to contribute to drug resistance [171]. Due to the avascularity or leaky vasculature of many solid tumors, drugs are often not able to penetrate into the hypoxic regions of the tumor resulting in drug resistance [171,172]. Spheroids represent an important model to mimic this in vitro, as they have areas of necrosis which mimic these areas of the tumor in vivo [108,173]. A study carried out by Däster et al [108], was able to show that treating cells with 5-FU resulted in different levels of sensitivity to the treatment depending on the stage of spheroid growth and size of hypoxic region (indicated by positive staining of HIF-1α. Once again, these studies show how 3D models are more representative of the in vivo environment then 2D cell culture models.

Similarly, treating 2D and 3D cultures of HCT116 cells with cisplatin results in different levels of sensitivity [134]. Moreover, using 3D models comprised of a variety of cells derived from a primary CRC tumor sample showed a resistance to 5-FU treatment, whereas the 2D model did not replicate this resistance, again confirming that this 3D model may be more representative of the in vivo model [174]. As mentioned previously in this review, CMS4 CRC is associated with high stromal infiltration [16]. Stromal cells have also been shown to mediate resistance to chemotherapy with subpopulations of MSCs contributing to immunosuppression as well as resistance to 5-FU [55,175]. This emphasizes the need to study multicellular models of CRC in order to further understand the role of stromal cells in late-stage CRC.

In the future, studies using microfluidic devices with advanced vasculature systems, incorporating stroma, may be used to study resistance to drugs such as cisplatin and other anti-angiogenic drugs, such as bevacizumab. Another future perspective for multicellular 3D models could be investigating synthetic lethal interactions within the TME. Synthetic lethal interactions are defined by two genetic mutations which, when combined, lead to cell death but individually do not [176,177]. For multicellular 3D models including stromal cells, it may be possible to use these models to co-target EGFR alongside other factors such as stromal cell factors that promote tumor growth. Analyzing cell viability in these 3D models may indicate which co-targeted genes have the biggest effect on the viability of different cells, thus identifying targets for future therapies for CRC.

The examples shown above demonstrate the use of 3D models in drug screening. Many of these models more closely represent the in vivo microenvironment of CRC by allowing cells to grow in a 3D environment and also include a variety of components present in the TME of CRC. Using cells from different lineages will provide a more replicative model for response to treatments and determining the cause for drug resistance which may result in new treatment options.

## 5. Conclusions

3D models are becoming increasingly important for the study of cellular interactions that take place in the TME of CRC in vivo. One of the key aims of these models is that they will reduce the use of animal models in the future. A reproducible multicellular 3D model is essential for assessing cellular interactions, progression of CRC, as well as drug discovery.

**Figure 6:**
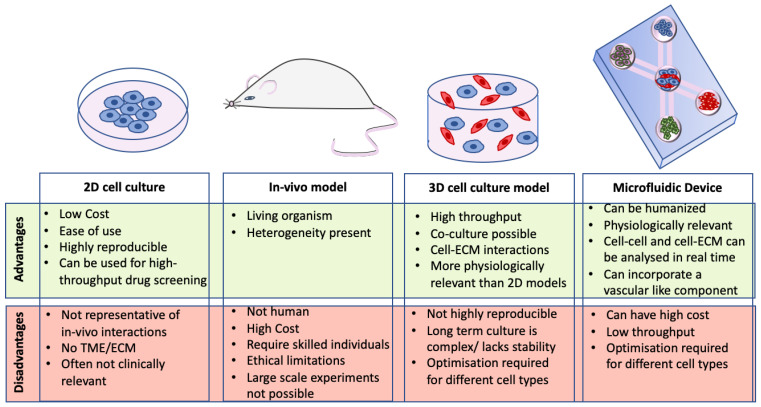
Advantages and disadvantages of CRC models. Each model has different attributes which are desirable for the ideal model of CRC. This figure summarises the major advantages and disadvantages of 2D cell culture models, 3D cell culture models, microfluidic devices, and in vivo models.

The ideal 3D model of CRC would recapitulate the main features of CRC TME including vasculature, stroma, immune cells and other components of the ECM present in vivo (e.g., collagen, laminin, or fibronectin) with the cancer cells to fully understand the TME as a whole. Furthermore, it should mimic the tissue stiffness of CRC in vivo and be easily reproducible with little variability. Development of reliable, reproducible assays is pivotal to the success of more complex 3D models. Each of the models mentioned in this review have different attributes which are advantageous in creating the ‘ideal’ model of CRC (Figure 6).

Currently, microfluidic devices appear to be one of the most representative models of CRC as they can incorporate a variety of cells, a vascular component, ECM proteins such as collagen (which can vary in stiffness), and are highly customizable for each model. These models require a lot of optimization but are very promising for studying cellular interactions in the TME of CRC. A hybrid model introducing spheroids into microfluidic devices could represent the way forward.

Overall, 3D models represent a promising tool for studying cellular and extracellular interactions between various cell types in CRC that dictate therapy responses, resistance and tumor progression. Therefore, 3D models promise to enable the testing of therapeutics, identifying resistance to specific therapies and studying the progression of CRC in a manner representative of the in vivo CRC TME. As well as this, these models have the potential to develop personalized patient 3D models which could ideally be used for analyzing therapeutics for these patients in the future. By combining a number of aspects from the variety of 3D models mentioned in this review, researchers may be able to bridge the gap between in vivo and in vitro models, creating a model that fully recapitulates CRC in vitro.

## Data Availability

Data sharing not applicable.

## Abbreviations

α-SMA	Alpha smooth muscle actin
CAFs	Cancer associated fibroblasts
CRC	Colorectal cancer
CMS	Consensus molecular subtype
CTCs	Circulating tumor cells
ECM	Extracellular matrix
EGFR	Epidermal growth factor receptor
EHS	Engelbreth–Holm–Swarm
FOXP3	Forkhead box P3
5-Fu	Fluorouracil
GelMA	Gel methacrylate
GM-CSF	Granulocyte macrophage-colony stimulating factor
HIF 1-α	Hypoxia inducible factor 1-α
iNOS	Inducible nitric oxide synthase
LGR5	Leucine rich repeat containing G-protein coupled receptor 5
LOX	Lysyl oxidase
MMP	Matrix metalloproteinase
MSCs	Mesenchymal stromal cells
NK	Natural Killer
NF-κB	Nucelar factor-kappa B
TGF-β	Transforming growth factor-beta
TME	Tumor microenvironment
PDMS	Polydimethylsiloxane
RGD	Arginylglycylaspartic acid
VEGFR	Vascular endothelial growth factor receptor
Wnt	Wingless-related integration site
